# Food safety governance information tool design in trust crisis - Analysis based on consumer trust perspective

**DOI:** 10.1016/j.heliyon.2023.e15866

**Published:** 2023-04-29

**Authors:** Meiying Chen, Shouxian Huang, Guoxing Huang, Qingqing Dang, Kai Li

**Affiliations:** a College of Economic and Management, Fujian Agriculture and Forestry University, Fuzhou 350002, China; b College of Anxi Tea, Fujian Agriculture and Forestry University, Anxi 362406,China; c College of Economics, Qufu Normal University, Qufu 273165, China

**Keywords:** Food safety, Crisis of confidence, Consumer trust, Willingness to pay, Principal component analysis method

## Abstract

In this paper, we use the method of principal-form analysis, based on 836 consumer survey data obtained from mobile Internet, and analyze the trust of current residents' tea consumption behavior on information content, presentation form, subject and other elements of information tools and their influence on the pollution-free certified products with the help of descriptive statistical analysis, KMO test and common factor extraction method. It was found that, firstly, the higher the trust of tea consumers in information content, the higher the additional willingness to pay; secondly, the form trust also significantly affects tea consumers' willingness to pay for pollution-free certified tea, and the specific cognitive information presentation form can effectively enhance tea consumers' willingness to pay; thirdly, there are significant differences in the trust of subjects, and enhancing the trust of industrial subjects helps to improve the pollution-free certified industrial The effect of trust of external subjects is not significant; fourth, the higher the tea consumers' care about the attributes of experienced products, the higher the degree of knowledge about the three products and one standard, and the higher the consumers' education, the higher they are willing to pay higher prices for traceable tea.

## 1 Introduction

In recent years, China has reported food safety incidents frequently. Information between the modern food industry and consumers is widely available. However, consumers have difficulty searching for various food safety information (Quan and zeng, 2013) [1]. Thus, the food market reputation mechanism has failed (Wu, 2012) [2]. The real risks and trust crisis in the food industry have become prominent food safety issues in China (Zhang et al., 2017; Xu, 2019) [3,4], especially in the media era. The spread of various rumors and false information is further exaggerated by people, impacting the food industry. Thus, the market is easy to fall into a dilemma caused by consumers’ lack of trust, the lack of forward incentives, and the lack of effective administrative supervision (Guo, 2020) [5].

After industry trust is damaged, the necessary market intervention of the government is the necessary conditions for market reputation reconstruction (Mo et al., 2016) [6]. The effective disclosure of food quality safety information is an important condition for rebuilding market reputation mechanisms (Zhang and Ke, 2002; Wu, 2013) [7,8]. Information tools^1^ have become the most basic and most important governance tools for food safety issues (Gong et al., 2013; Gao and Liao, 2015) [9,10] On the one hand, given that information is asymmetrical and is the root cause of food safety issues (Caswell, 1992; Wang and Sun, 2002) [11,12] and that information disclosure is the best response to information asymmetry (Ogus, 2004) [13], real complete information is the foundation of food risk management. On the other hand, compared with other regulatory tools, the information tool applies to the food market with low market concentration and high information asymmetry and is low in implementation (Ying and Tu, 2010) [14]. Although the Chinese government has introduced and is constantly improving the standardization of compulsory information disclosure, the enthusiasm and initiative of various subjects, industry associations, and news media are constantly enhanced. However, whether it is mandatory disclosure of government information or information reported by enterprises and other entities, it belongs to the information supply category. To improve the effectiveness of information tools, it is also necessary to take fully into account the information demand side of food quality and safety information, i.e., the characteristics of consumers' information demands and the degree of information trust. Defining information disclosure content according to the consumer information needs and trust characteristics (Gong et al., 2013) [9]and determining the right information disclosure and regulatory platform are needed to improve the effectiveness of the information tool (Ying and Tu, 2010) [14]. In the particular environment of the food industry crisis and consumer over-panic, effective assessment of consumers' trust in information tools is crucial for optimizing the design of information tools. Anxi Tieguanyin, once a high-end tea in consumers' mouths, has made a great contribution to the overall economic and social development of Anxi County, placing first in "China's 100 key tea producing counties” and “Top 10 National Expo teas”. However, under the influence of various reports of excess pesticide residue and added flavourings, the reputation of Anxi Tieguanyin started to decline, and the development of the industry has been hindered and is still in a period of decline. In this study, we take as an example Anxi Tieguanyin, which has been plagued by rumors of food safety incidents and has fallen into a market trough, and test the impact of different information tools on consumer trust from the perspective of consumers' trust in various components of information tools, with the aim of providing a benchmark for rebuilding consumer confidence in certification and promoting the establishment of a market mechanism for high quality and high price agricultural products.

## 2 Literature review

### 2.1 The roots of food safety problems and the form of food industry trust

Nelson (1970) categorizes goods into search goods, experience goods, and trust goods,^2^ where there is asymmetric information between producers and consumers for both the experience goods and the trust goods [15]. Food safety risk factors, such as microbial pollution, heavy metal pollution, and farmers often have experience or trust characteristics. Information asymmetry has different levels. Asymmetrical information in the experience market can be solved by reputational economic incentives and multiple purchase experiences, but the trust market requires third parties to provide a signal transmission mechanism (Wang and Sun, 2002; Caswell and Mojduszka, 1996) [12,16]. Converting food quality trust to search characteristics to eliminate the information asymmetry of the food market is necessary. For food search products, information asymmetry is not obvious because consumers can master information. Furthermore, production operators can pass brand certification or implement the traceability system to deliver complete information to consumers and gain their full trust (Wei and Gao, 2017) [17].

### 2.2 Research on coping during the food industry's trust crisis and reconstruction

The efficient provision of quality and safety information is seen as a fundamental step in addressing the problem of food market failures. Food safety traceability and certification systems are important forms of quality and safety signals, and they are important vehicles for the disclosure of information by actors in the food industry. Willingness to pay for products with different quality and security information such as traceability, guaranteed quality and safety and animal welfare, the greater the disclosure of information content, the higher consumers' willingness to pay (Dickinson and Bailey, 2002; Wu et al., 2014; Yang et al., 2016). [[18], [19], [20]]. In addition to the amount of content, information can be positive or negative, and information diffusion channels affect information trust. Ni et al. (2018) believes that the combination of negative information element and public relational information element can be disclosed to avoid negative information [21]. Zhang (2017) surveyed food safety information demand for Beijing consumers and consumers' preferred official channel information [22]. Xu et al. (2017) believes that information tools can directly affect the public's consumption tendency, operators' substant interests, and social belongings and enhance the level of trust in food safety administrative regulations [23]. Scott et al. (2014) [24] believes that community-supported agriculture (CSA) can build consumer trust because CSA can provide scenario embedding (Meyer et al., 2012) [25]. Consumers can clarify the food source, enable consumers and producers to communicate with interactive bridges (Chen, 2015) [26], and reduce risk and uncertainty through frequent interaction. Frewer et al. (2003) discovered that European consumers (from Denmark, Germany, Italy, and the UK) have found that information comes from the same or different information publishers and that all information portfolios do not lead to systemic changes in consumer attitudes [27]. Dean and Shepherd (2007) provide an experimental participant's message for two different sources. Such information is about genetically modified food risk, income, consistency, or conflict [28]. Studies have found that consistent information reduces consumers' perceived risk. Zhang et al. (2016) indicate that the credibility of the distribution subject has a significant difference in the impact of consumer attitude [29]. The information source of high credibility is likely to affect the attitude of consumers on genetically modified foods. Verbeke and Ward (2005) found that, compared with third-party certification, Belgian consumers prefer to be trained by the government and industry associations [30]. Umberger et al. (2003) found that 73% of respondents would pay more than 11%–24% for traceable beef with origin information [31]. Hobbs (2002) pointed out that Canadian consumers are willing to have additional animal welfare and quality and safety guarantees for beef and pork sandwiches for $1.12 Canadian dollars [32]. Ortega et al. (2014) [33], Yi et al. (2013) [34], and Zhang et al. (2014) [35] investigated China's consumers and found similar conclusions. Zhang et al. (2013) has the highest payment of the government's certification [36]. The certification of third-party institutions is complementary to government certification. Yin et al. (2015) shows that consumers are willing to pay 5.16 yuan for organic labels [37]. Ying et al. (2016) discovers that consumers are willing to pay more than 3.2 yuan/kg for traceable pork with quality management system certification and more than 2.9 yuan/kg for the traceable pork with the supply chain traceability information [38]. Domestic and international studies have found that consumers have an additional willingness to pay for products that are certified or traceable.2.3 Specialty and trust reconstruction of food safety governance.

In China, given the low market concentration, the food industry has many chain lengths, resulting in non-satisfactory conditions of the generation and stability of the trust mechanism (Tao and Zhou, 2012) [39]. Food safety is difficult to translate into trusted products (Wu, 2012; Zhou et al., 2004) [2,40]. From the perspective of domestic certification label payment and the purchase behavior of certified products, domestic consumers have little trust in domestic quality and safety signals. Zhang et al. (2014) discovers that safety certification can eliminate information between producers and consumers to a certain extent and change consumer expectations and payment of food safety [35]. Zhou et al. (2008) analyzed consumers' purchase behavior to trace the label beef and found that only 50.73% of the respondents buy [41]. Wang et al. (2009) found that only 30.1% of the respondents are willing to pay higher than 10% in Shandong, Beijing, Zhejiang, Wang Feng, and other regions [42]. Jiang et al. (2013) estimation results for the willingness to pay for the port of Hong Kong show that consumers trust the “Forport Standard” and that the average premium level is approximately 44%, which is far higher than the premium level of other domestic certification standard products from some domestic scholars [43]. Yi et al. (2019) found that the knowledge of food safety certification and food traceability system can significantly improve the payment of consumers on organic identity and traceability information identification, and the willingness of the green logo and pollution-free logo have low impact [44]. Zhou (2020) believes that the government publicly impacts unqualified product quality information on consumers and overall industries, which may change consumers' willingness to pay for quality products, and indirectly affects producers' HACCP certification [45]. Wu et al. (2014) found that consumers were more preferred in China's third-party institutions because frequent pork quality safety incidents made consumers question the government's credibility [46]. Li et al. (2015) conducted a survey of 752 consumers in Jinan and other cities and found that consumers are considerably less willing to pay for organic labels in China compared to other areas such as the EU [47]. Yue et al. (2017) found that consumers' trust in product information and reputation signals is lower than quality assurance and service quality signals [48]. Consumers' domestic food safety certification is lower than other quality signals, indicating that domestic certification has a certain degree of failure (He et al., 2008; Zhou et al., 2008; He et al., 2017) [49,50]. Quality safety signal failure is the root cause of consumers' cognition. The signal is not effectively judged and identified; the lack of seriousness of the producer and the seriousness of the certification process have not reflected the real quality and producer of agricultural products (Wang and Gu, 2012) [51].

Much of the existing food safety literature is concerned with the root causes of the problem and forms of trust in the food industry, in response to the food industry crisis of trust and trust reconstruction and so on. Special features of food safety governance and special features of trust reconstruction in China have also been investigated as a result. A very useful reference for the study of this article is the existing literature. It is not hard to see, however, that existing studies still suffer from the following shortcomings: First, existing research literature rarely analyses tea consumers' food safety governance from a consumer safety information trust perspective. Secondly, the existing international literature on traceable attributes of food information and their hierarchical frameworks is not consistent with the Chinese context. Information on animal welfare, for example, is a common concern among Western consumers, but information on animal welfare is not a common concern among Chinese consumers, at least at this time. Thirdly, there is a relative paucity of research in the existing literature on food safety governance information tools. Consequently, in the current special environment in which the degree of food safety certification is low and the public speaks about food, the aim of this paper is to explore the impact of different elements of information tools on consumer trust from the perspective of the food safety quality and safety information trust of Tieguanyin tea consumers, which is important in reconstructing consumer trust.

## 3 Study design

### 3.1 Research methods

#### 3.1.1 Principal component analysis

Principal component analysis (PCA), which was introduced and used in the early 20th century, is now the most common research method for studying consumer trust. The method puts different kinds of influencing factors together and uniformly screens the intensity of the factors, eliminating highly correlated influencing factors while ensuring a small amount of information loss, which can achieve the purpose of dimensionality reduction while retaining important information [52], and the basic principles of the method are as follows:

First, data standardization. Standardization of the influence factor data, due to the different measures of different contents of tea consumer trust, the variables cannot be directly compared with each other, it is necessary to dimensionless the variable data, and the variables can be compared only after standardization. Assuming that n samples need to be analyzed, the p-dimensional random vector X is expressed as:

(1) $$X_{i}=\left(\begin{array}{cccc} X_{1} & X_{2} & \cdots & X_{p} \end{array}\right)\;T$$

where $X_{i}$ is the following expression:

(2) $$X_{i}=\left(\begin{array}{cccc} x_{1i} & x_{2i} & \cdots & x_{ni} \end{array}\right)\;ˆ\left(T\right),\left(\begin{array}{c} i=1,2,\cdots , \end{array}n,n>p\right)$$

The sample matrix is constructed for the analyzed variables and standardized by the formula:

(3) $$Z_{ij}=\frac{X_{ij}-\begin{array}{c} {\bar{X}}_{j} \end{array}}{S_{j}},\begin{array}{c} i=1,2,\cdots \text{,} \end{array}n;j=1,2,\cdots ,p$$

The expressions for ${\bar{X}}_{j}$ and $S_{j}$ in the above equation take the form:

(4) $${\bar{X}}_{j}={\sum_{i=1}^{n}\frac{X_{ij}}{n},{S_{j}}^{2}=\sum_{i=1}^{n}(X_{ij}-{\bar{X}}_{j})}^{2}/\left(n-1\right)$$

Next, correlation analysis was performed. After the standardization of variable data for correlation analysis, and the correlation coefficient matrix was derived. There are many variables in the influencing factors of tea consumer trust that may be highly correlated, and such variables with relatively large correlation will appear as covariance in the regression analysis, using the formula:

(5) $$R={\left[\begin{array}{c} R_{ij} \end{array}\right]}_{p\times p}=Z^{T}Z\;/\left(n-1\right)$$

Find the correlation coefficient matrix R, excluding some variables with too large correlation, where Z denotes the normalization matrix in the above equation, and $\begin{array}{c} R_{ij} \end{array}$ is expressed in the form of:

(6) $$R_{ij}=\sum z_{ki}\times z_{kj}/\left(n-1\right)$$

Third, the main impact factors are determined. The eigenvalues, eigenvectors and contribution of the correlation coefficient matrix are calculated to finally determine which are the main influencing factors. The eigenvalues are calculated as:

(7) $$\left|\begin{array}{c} R-{\lambda I}_{P} \end{array}\right|=0$$

From the above equation, the p characteristic roots can be derived, and the main impact factor is determined according to the following equation:

(8) $$\sum_{j=1}^{m}\lambda_{j}/\sum_{j=1}^{p}\lambda_{j}\geq 0,j=1,2,\cdots ,m$$

The variables with a contribution of 85% and above are identified as main factors and the generalized equation is solved:

(9) $$Rb=\lambda_{j}b$$

The eigenvector ${\begin{array}{c} b_{j} \end{array}}^{0}$ is derived, and then the following principal component formula is used to find the main influencing factors of tea consumer trust:

(10) $$\begin{array}{c} U_{ij}=\begin{array}{c} {{{Z_{i}}^{T}b}_{j}}^{0} \end{array} \end{array},j=1,2,\cdots ,m$$

In the above formula $U_{1}$ is the first principal component, $U_{2}$ is the second principal component, and so on $U_{P}$ is the pth principal component. The final evaluation value is obtained by weighting and summing the above m principal components with the variance contribution rate as the weight, and the comprehensive evaluation is performed based on the evaluation value.

#### 3.1.2 KMO test and Bartlett's sphericity test

Before conducting principal component analysis, we have to determine whether the data are suitable for principal component analysis, and this time the KMO test and Bartlett's spherical test are used [53].

(1) KMO.

It is used to check the bias correlation between variables and takes a value between 0 and 1. The closer the KMO value is to 1, the stronger the bias correlation between variables is and the better the factor effect is, the more suitable it is for principal component analysis. a KMO value above 0.9 is extremely suitable, above 0.8 is suitable, above 0.7 is okay, above 0.6 is barely okay, above 0.5 is not suitable, and below 0.5 is very unsuitable. In practice, above 0.7, the results are better; when it is below 0.5, it is not suitable for principal component analysis [54].

(2) Bartlett's spherical test.

Used to determine whether the correlation matrix is a unit array, i.e., whether the variables are strongly correlated. p < 0.05>0.05 obeys the sphericity test, and the variables are independent of each other and cannot be subjected to principal component analysis [54].

#### 3.1.3 Complementary double logarithm model

The complementary double logit model is a multiple regression analysis model. The complementary double logit model is based on the assumption that the error distribution obeys the extreme value distribution, and is applicable to asymmetric binary discrete data, and can be used to study the effects of solving multiple factors. When the variables take values in the range of 0 to infinity, the complementary double logit model can effectively adjust the data and simplify the analysis and interpretation of results. Traditional regression models assume that the model error distribution is logistic and normal, respectively, while some cases errors do not meet such assumptions, and their error distribution is an asymmetric extreme value distribution or Gumbel distribution. Such as the highest water level of a river in a certain year such an extreme value, then need to seek a more effective model, then need to use the complementary double logarithmic model for analysis [55].

The general form of the model is:

(11) $$\log\left\{-\log\left[1-P\left(Y\leq j\right)\right]\right\}=a_{j}+\beta^{,}X\text{,}$$

From this, the expression to find the probability can be obtained as

where: $a_{j}$ is the portion of the explanatory variables that cannot be explained; $\beta^{,}$ is the vector of parameter composition. When there is only one independent variable and when X = $x_{i}$ (i = 1,2), there is the property:

(12) $$P\left(Y>j-|x_{1}\right)=P\left(Y>j-|x_{2}\right)\exp\left[\beta \left(x_{1}-x_{2}\right)\right]\text{.}$$

The regression coefficients for the respective variables are usually estimated using the maximum likelihood method. Since the complementary double logarithmic function is strictly concave and the Hessian matrix is negative, parameter estimates are obtained by the Newton-Raphson method [56], and the parameters are tested, as well as the model goodness-of-fit test [57].

(The study protocol was approved by the Ethics Review Committee of Fujian Agriculture and Forestry University. Written informed consent was obtained from all study participants, and all procedures were performed in accordance with the Declaration of Helsinki and relevant Chinese policies).

### 3.2 Variable selection and data sources

#### 3.2.1 Variable selection

The function of trust is reflected in the establishment of a number of simplifying mechanisms for the complex natural and social environment in which human beings live. The effective functioning of the market for trustworthy goods depends not only on peer competition, but also on an effective regulatory system and public trust in the regulatory system (Wang et al., 2014). [58]. The presence of food safety experience and trust characteristics makes trust particularly important in the food market. Drawing on Luhmann's (1979) classification criteria, this study classifies tea consumer trust into two categories: subject trust and institutional trust [59]. The specific indicators for each variable are shown in Table 1.

(1) Subject Trust

**Table 1:** Specific indicators for each variable.

Variables	Specific indicator
Industry subject trust	Farmer trust
	Corporate trust
External regulatory subjects trust	Industry association Trust
	News media trust
	Sales platform trust
	Government trust
	Expert trust
Information form trust	Text trust
	Picture trust
	Video trust
	Commentary trust
	Real-time interactive trust
Information content trust	Environmental information trust
	Input information trust
	Certification information trust
	Sampling information trust
	Credit information trust
	Third-party information trust
	Sales platform information trust

Interpersonal trust is the emotion between people often in acquaintance circles, such as homes and neighbors, and presents strong and weak differences similar to “differential pattern” (Luo and Ye, 2006) [60]. With the integration of Internet technology and the food industry, the Internet age continues to deepen, and food production and sales models continue to innovate. Vegetables, community support agriculture, network direct sales, and other direct consumers and producers’ short-chain food marketing models have emerged. Thus, in the traditional sense, interpersonal trust is also established in the food industry chain (Shuai, 2013; Xu and Zhou, 2016) [61,62]. Considering the difference between tea consumers' trust in industry subjects and external subjects, this study divides the subject trust into industry subject trust and external regulatory subject trust.

First, the industry subject trust. It mainly includes farmers and food production and management enterprises trust. As food producers, farmers' agricultural production is characterized by multiple goal attributes. In addition to market economic benefits, ensuring food safety, reducing labor dependence and reducing production risks are also the main goals pursued in farmers' production. The production and management entities are the first responsible for food safety. In the Internet era, food marketing models such as vegetable groups and agricultural-consumer docking have made it possible to establish interpersonal trust between production and management agents and consumers, and this relationship helps transform systemic trust into personal trust, an effective way to increase trust in food safety (Xu and Zhou, 2016) [62].

Second, the external regulatory subjects trust. Including the trust of government and the subjects of social surveillance. Trust is a precondition and a key factor for market confidence. Fushan (2001) believes that China is a low-trip country, introducing government strength to eliminate the barrier construction of market trust in the market [63]. Luo et al. (2012) studies the relationship between China's and Taiwan's high-tech outsourcing enterprises and finds that the stability of the government and its policies directly affected the behavior of both sides of the transaction [64]. Based on the case study of Shenzhen food safety society, Xie (2017) indicated that the new self-organized construction regardless of authority is intervention and that self-organizational construction can be successful [65]. Subject trust is social control. Including industry association trust, third-party trust, news media trust, and expert and scholar trust. In China's distrust of the food market, the government is limited by administrative resources, which makes it difficult to conduct all-round monitoring and governance. To do this, social resources must be mobilized and leveraged and food safety supervision must be implemented with social supervision as an important aid to reduce the difficulty of government supervision and break through the dilemma of limited administrative resources. Fundamentally addressing the food safety crisis and the problem of inefficient administrative oversight, there is a need to mobilise and utilise social surveillance resources such as industry associations, certifiers, the press and consumers. Social supervision has the potential to effectively expand the coverage of food safety supervision and to break the administrative resource constraints faced by a single administrative supervision. In addition, social supervision such as industry associations, certifiers, and the press may play a complementary role to administrative supervision, with the former imposing social sanctions on illegal firms (Zhang et al., 2017). [3].

(2) Institutional trust

The system is the key to rebuilding trust. The system is the basis of trust. System trust is often dependent on the legal, political, and institutional environment, which is trust in “non-interpersonal” relationships (Zhang and Tang, 2005) [66]. Trust is often the result of people's rational choices (Coleman 1990; Hardin, 1993,1999, 2000) [[67], [68], [69], [70]]. Kreps (1986) [71], Fudenberg and Tirole (1992) [72], Zhang et al. (2002) [73] uses a repetitive game model to prove that the long-term interests under repeat game will result in trust. As an important game rule, information structure has a direct impact on the game results. In terms of the food safety game, information is a “double-edged sword.” On the one hand, information is an important prerequisite for the role of the reputation mechanism. No efficient information transfer is observed, and reputation mechanism cannot work. On the other hand, frequent transfer of negative information will damage the public's trust in the economic and social order, which will affect the effectiveness of the reputation mechanism and may trigger a series of chain reactions, eventually leading to a trust crisis. The source of information, the content of information and the form of information are all important factors affecting the effectiveness of the information disclosure system.

First, the information source. Some scholars believe that traditional authoritative media is the key to rebuilding public trust. Scholars such as (Utz, Schultz and Glocka, 2013) [74], who studied the Fukushima disaster, found that, although crisis communication through social media is favorable and effective, unfavorable secondary crisis is spread. Nevertheless, traditional media communication still plays an important role and helps in rebuilding public trust. The public tends to accept a defensive, supportive, or circumventive crisis. However, studies have been conducted after a crisis occurred. Urgent information needs to be publicized in new media to make the public obtain immediate and in-depth information. Social media plays an increasingly important role during a crisis. Taylor and Botan (2005) believes that emerging communication channels provide opportunities to communicate with the public to compete with existing traditional communication channels [75]. Further, if a new medium or source of trust offers information to people and shoulders the ethical responsibility, the new information source will receive the same public trust as traditional trust. Despite effective arguments regarding information, the effective match of communication channels has become a consensus on trust construction.

Second, the content and form of information. Content information is primarily represented by the information involved in the link and the amount of content, and the form is split between the traditional text, image, video form and whether it has interactive feedback, live or otherwise. Botan and Frey (1983) say that source credibility is an essential factor in constructing public trust. Source credulous reliability depends on the content and form of information [76]. Shao et al. (2020) believes that specific content and form of information disclosure should meet the information demand for social organizations to regular management activities and normalized governance mechanisms [77]. Information portfolio generally refers to the classification combinations of all kinds of information publishing subjects, information content, and information channels (Zhang et al., 2020) [78]. Thus, this study will further divide system trust into content information and presented information.

#### 3.2.2 Data sources

Tea has a long history of consumption in China, and is also an important representative product of healthy food consumption. Anxi Tieguanyin, as the happy tea in people's mouth, the total output value of tea-related industries in Anxi County in 2011 was 9.2 billion yuan, and the population benefited from tea reached more than 800,000 people, and the tea industry made great contributions to the overall economic and social development of Anxi County. However, after 2011, various kinds of pesticide residue exceeded the standard and added flavoring reports, the reputation of Anxi Tieguanyin began to decline, and the development of the industry has been hampered and is still in the doldrums. After the pesticide residue and flavoring incident, coupled with the network media boiling hype, resulting in consumer psychological panic and resistance, affecting the consumer's desire to consume Anxi Tieguanyin, customers are also increasingly cautious about buying tea. Due to the impact of excessive pesticide residues and flavoring incident, Anxi County Government in recent years launched a tea industry development “six unified” development strategy, namely, the county to implement a unified agricultural supply, unified pest guidance and prevention, unified technical training, unified tea brand, unified quality management and unified market sales Wei, strong support for tea professional Cooperative development, to help achieve the second take-off of Anxi Tieguanyin.

The research data came from a survey of residents' Tieguanyin tea consumption behavior conducted through the mobile Internet network in 2018. A total of 900 questionnaires were returned, and 836 valid questionnaires were obtained by removing key questions missing or obviously wrong questionnaires, with an effective rate of 92.89%. The questionnaires were distributed through the Internet because it is easier to access various types of information through cell phone networks and computer networks, and this information increases the consumer's food safety knowledge base on the one hand, but on the other hand, it also has an impact on the consumer's inherent knowledge to a certain extent, thus better demonstrating the influence of the information trust element on the consumer's purchase decision of certified products.

Since the survey relied on the Internet, the respondents were relatively young, highly educated, and familiar with online shopping in general. First, in terms of the respondents' education level, the average education level of the sample was college or above, and the largest number of respondents (48.2%) had a bachelor's degree or above. Second, in terms of age, the average age of the respondents was 33.29 years old. Third, in terms of income, the annual household income of the respondents ranged from 60,000 to 150,000 RMB, with the highest percentage of 60,000 to 100,000 RMB (21.8%), followed by 100,000 to 150,000 RMB (20.1%). Fourth, 58% of the respondents cited online channels as their main purchase channel.

### 3.3 Descriptive statistical analysis

In terms of respondents' attitudes towards food safety, the average score for food safety concern was 4.16, with the majority of respondents attaching great importance to food safety. However, there were significant differences between the three categories of food safety-related attributes: trustworthy attributes (including pesticide residue content, heavy metal content, and microbial content) received the most attention, with an average score of 4.4, search attributes (e.g., appearance, color, fragmentation, aroma, packaging) scored 4.04, and experience attributes (e.g., brand) scored 3.57. The specific scores for the three categories of attributes are shown in Table 2. Trust goods imply that it is difficult to obtain effective information even after consumption is completed, and thus trust goods have the most serious information asymmetry between producers and consumers, so traceability, which aims to provide relevant information and reduce the degree of information asymmetry, is rightly favored by consumers who are concerned about the trust goods attribute.

**Table 2:** Consumers’ differences in three types of properties for food safety.

Food safety attribute	Specific indicator	Ranges	Variable explanation	Average value	Variance
Trust	Farming content	1–5	1 - nothing, 2 - not tight, 3 - general, 4 - more important, 5 - very important	4.41	0.920
	Heavy metal content	1–5		4.42	0.902
	Microbial content	1–5		4.38	0.919
Search	Shape	1–5		3.9	0.873
	Abcrosis	1–5		3.97	0.847
	Aroma	1–5		4.24	0.850
	Outer packaging	1–5		4.05	0.707
Experience	Brand	1–5		3.57	0.934

From the consumers' evaluation of the current situation of quality and safety, the proportion of consumers who think it is now very unsafe and not very safe is only 4.1%, and those who think it is average is 12.9.83% of consumers think the current situation of tea quality and safety is good (relatively safe 45.6%, very safe 37.4%). In terms of tea drinking frequency, 84% of the respondents drink at least once a week, but only 1.2% of the respondents drink every day. From the respondents' familiarity with the three products and one standard, only 5.6% said they did not know at all, and only 6.6% said they knew very well, and more than half of the consumers said they knew a little (50.8%), which means that consumers do not know much about the three products and one standard, and the relevant quality and safety status evaluation and basic information are shown in Table 3.

**Table 3:** Consumer evaluation of the current state of quality and safety and basic information.

	Ranges	Variable explanation	Average value	Variance
Food safety attitude	1–5	1 - don't care at all, 2 - not concerned, 3 - general, 4 - more concerned, 5- very concerned	4.16	0.678
Status quotes	1–5	1 - very unsafe, 2 - not too safe, 3 - general, 4 - safe, 5 - very safe	2.45	0.895
Tea frequency	1–5	1 - almost no drink, 2 – once a week, 3 - a week, 4 – at least twice a week; 5 - Drink every day	2.96	1.711
Online-oriented	0–1	0 - Offline channels primarily, 1 - Online shopping primarily	0.58	0.244
Certification	1–5	1 – don't know at all, 2 - have heard of it, 3 - know it, 4 - is more familiar, 5 – is knowledgeable	3.04	0.857
Education level	1–4	1 - junior high school and below; 2 - high school, technical school, and secondary school; 3 -college; 4 - bachelor	3.11	1.022
Family annual income	1–6	1- Annual household income of RMB 30,000 and below; 2- RMB 30,000–60,000; 3- RMB 60,000–100,000; 4- RMB 100,000–150,000; 5- RMB 150,000–200,000; 6- More than RMB 200,000	3.50	2.574

## 4 Empirical analysis

### 4.1 Principal component analysis

#### 4.1.1 KMO test and Bartlett's test

First, the data for each variable are standardized, and since the variables expressing the trust factors of tea consumers may be somewhat correlated, a direct regression analysis would lead to inaccurate results. Therefore, before using principal component analysis for information enrichment studies, it is necessary to analyze whether the study data are suitable for principal component analysis.

KMO test is mainly used to check the correlation and bias correlation between variables, and this paper can test the data situation of principal component extraction. Generally speaking, the KMO test coefficients are distributed between 0 and 1. The closer the coefficient is to 1, the stronger the correlation between variables and the weaker the bias correlation, the better the effect of factor analysis. If the coefficient value is greater than 0.6, the sample is considered to meet the requirement of reasonable data structure. However, previous scholars generally believe that the results of principal component analysis have better practicality only when the KMO test coefficient value is greater than 0.8. In this study, the KMO coefficient value reaches 0.913, as shown in Table 4, indicating that the data structure is good and the results of principal component analysis have good utility. the statistic of Bartlett's test is obtained based on the determinant of the correlation coefficient matrix, and if the value is large and its corresponding companion probability value is less than the significance level in the user's mind, then the null hypothesis should be rejected and the correlation coefficient matrix is considered not likely to be a unit array, i.e., there is correlation between the original variables and it is suitable for principal component analysis; on the contrary, if the statistic is relatively small and its corresponding probability of companionship is greater than the significance level, the null hypothesis cannot be rejected and the correlation coefficient matrix is likely to be a unit array and it is not suitable for factor analysis. In this study, the *P*-value of Bartlett's test was less than 0.001, and the null hypothesis was rejected, which means that the study data were considered to be ready for principal component extraction.

**Table 4:** KMO and Bartlett's test.

KMO and Bartlett's test		
KMO Sampling		0.913
Bartlett's Spell Test	Approximate card	8650.926
	Freedom	171
	Significance	0.000

The results of the KMO test for the analysis of individual variables were also distributed between 0 and 1. If the coefficient was greater than 0.5, the individual variables were considered to meet the requirements; if the coefficient was greater than 0.8, the individual variables were considered to have good results. In this study, the KMO test results for any of the variables were greater than 0.5, which means that the results for each variable were average but still met the requirements. The results of the KMO tests for each variable are shown in Table 5.

**Table 5:** KMO test results.

Poor pastoral		
	Initial	Extract
Environmental information	1.000	0.702
Put into information	1.000	0.723
Certification Information	1.000	0.711
Sampling information	1.000	0.679
Credit information	1.000	0.547
Third party information	1.000	0.678
Platform information	1.000	0.523
Farmer	1.000	0.780
Enterprise	1.000	0.737
Industry association	1.000	0.683
News media	1.000	0.534
Sales platform	1.000	0.523
Government	1.000	0.754
expert	1.000	0.691
Writing	1.000	0.687
Image	1.000	0.747
Video	1.000	0.719
Comment	1.000	0.548
Real-time interaction	1.000	0.575

Extraction method: principal component analysis.

#### 4.1.2 Determining the number of principal components

Once the data were determined to be ready for principal component analysis, the next step was to determine the number of principal components. In this paper, the number of selected principal components is determined with the help of SPSS software. There is no precise quantitative method for determining the number of components, but a common method is to determine the number of components with the help of three criteria. One is the eigenvalue criterion, the second is the gravel plot test criterion, and the third is the expertise judgment method. The eigenvalue criterion is to select the principal components with eigenvalues greater than or equal to 1 as the initial components and discard the principal components with eigenvalues less than 1. The criterion of gravel plot test is to draw a line graph of the change of eigenvalues with the number of components according to the order in which the components are extracted, and to judge the number of components according to the shape of the graph. The characteristic of the line graph is from high to low, steep and then flat, and finally almost a straight line. The point before the curve starts to flatten is considered to be the maximum number of components extracted. The expertise judgment method is a subjective judgment of the number of components in combination with one's own expertise situation. This part uses the eigenroot value judgment method.

The variance explanation rate table is mainly used to determine how many principal components are appropriate to extract. The variance explanation rate and the cumulative variance explanation rate of each principal component are also used. The larger the variance interpretation rate is, the more the principal components contain the information of the original data. The analysis of the extracted principal components and the amount of information extracted from the principal components can be seen from Table 6: four principal components were extracted from the principal component analysis, and the root values were all greater than 1. The variance explained by these four principal components were 39.844%, 13.392%, 7.162%, and 5.605%, and the cumulative variance explained by 66.002%. In addition, their corresponding weighted variance explanations, i.e., weights, are in order: 39.844/66.002 = 60.37%; 13.392/66.002 = 22.32%; 7.162/66.002 = 10.85%; and 5.605/66.002 = 8.49%.

**Table 6:** Common consection test.

Total variance explanation									
Ingredient	Initial feature value			Extract the local			Rotating load square		
	Total	Voltage percentage	Cumulative%	Total	Voltage percentage	Cumulative%	Total	Voltage percentage	Cumulative%
1	7.570	39.844	39.844	7.570	39.844	39.844	3.829	20.155	20.155
2	2.544	13.392	53.236	2.544	13.392	53.236	3.679	19.363	39.518
3	1.361	7.162	60.398	1.361	7.162	60.398	3.453	18.172	57.690
4	1.065	5.605	66.002	1.065	5.605	66.002	1.579	8.312	66.002
5	0.807	4.245	70.248						
6	0.722	3.799	74.047						
7	0.610	3.210	77.257						
8	0.574	3.021	80.278						
9	0.477	2.511	82.789						
10	0.431	2.267	85.056						
11	0.424	2.231	87.287						
12	0.413	2.174	89.461						
13	0.355	1.868	91.329						
14	0.328	1.724	93.053						
15	0.315	1.657	94.710						
16	0.288	1.518	96.229						
17	0.270	1.420	97.649						
18	0.240	1.263	98.911						
19	0.207	1.089	100.000						

Extraction method: principal component analysis.

#### 4.1.3 Extraction of principal components

The number of principal components has been determined, and the loading coefficient matrix was obtained after analysis as shown in Table 7. The table of loading coefficients, mainly shows the information extraction of the principal components for the study items and the correspondence between the principal components and the study items. The common degree represents the amount of information that can be extracted from a question item, and the higher the common degree indicates that the index can be explained by the principal components to a higher extent and the more information is extracted. In summary, this paper chooses to extract the top four principal components, which reflect information content, information form, external subject, and industry subject, respectively.

**Table 7:** Common time test.

Rotate ingredient matrix a				
	Ingredient			
	1	2	3	4
Investment information	0.823			
Environmental information	0.807			
Certification information	0.782			
Sampling information	0.672		0.377	
Third party information	0.653	0.321	0.385	
Credit information	0.612	0.371		
Image		0.825		
Video		0.790		
Writing		0.751		
Comment		0.715		
Real time	0.392	0.608		
Platform information	0.403	0.574		
Government			0.837	
Expert			0.799	
Industry association			0.726	0.337
News media			0.659	
Sales platform			0.654	
Farmer				0.860
Enterprise			0.468	0.708

Extraction method: principal component analysis.Rotation method: Caesar normalization maximum variance methoda.

a Rotation has converged after six iterations.

It is worth noting that after the four principal components are extracted, there are two principal components explaining the same variable, including the first and second principal components explaining both third-party information, credit information, real-time and platform information. The first two are because third-party information and credit information are often presented in traditional forms such as text and graphics, and thus there is a strong correlation between information content and information form, and because the first principal component has a stronger explanatory power and is closer to the classification of information content, the two variables are assigned to the first principal component. The latter two variables, on the other hand, are classified into the second principal component because both real-time and platform information are not a single information content or form, but contain different forms and contents of information, according to the previous classification criteria. The first principal component and the third principal component jointly explain the sampling information and third-party information, which is because the sampling information and third-party information are usually provided by the government and the third party respectively, and thus there is a strong correlation between the information content and the information providing subject, and these two variables are assigned to the first principal component with reference to the aforementioned criteria. The third principal component and the fourth principal component jointly explain the industry associations and enterprises. Although the third and fourth principal components are both about the information providing subjects, the third principal component is mainly the third-party information providing subjects, while the fourth principal component is the farmers and enterprises, which belong to the practicing subjects. Although the current industry association belongs to the third-party organization and is a bridge linking the government and the industry, the industry association is also participated by various enterprises, so there is a strong correlation between the two. Also based on the aforementioned division criteria, the industry association is classified as a third-party information subject and the enterprise is classified as an industrial information subject.

#### 4.1.4 Results of principal component analysis

The higher the composite score of principal components means the stronger the trust of tea consumers in information tools, and the results of principal component analysis in this paper are shown in Table 8. Consumers have the highest level of trust in information, reflecting consumers’ knowledge confidence in their food safety. From specific content, consumers are willing to believe environmental information, sampling information, and input information (4, 3.99, 3.97); certification information and third party information (3.9 and 3.86); and finally subject credit information and platform information (3.67 and 3.5). From the analysis results of Liu et al. (2011) [79], Wen and Liu (2012) [80], and Zhu and Hong (2014) [81]on food safety events in China, it is clear that the irregular use of inputs in processing and production, and environmental pollution in origin are precisely the most important sources of food safety risks in China at present. Therefore, consumers, especially young consumers, have accurate understanding of the source of food safety risks in China.

**Table 8:** Table of principal component analysis of consumer trust.

	Specific indicator	Mean	Standard deviation
Industry subject trust	Farmer trust	3.23	0.86
	Corporate trust	3.04	0.82
External subject trust	Industry association Trust	3.43	0.93
	News media trust	3.47	0.88
	Sales platform trust	3.39	0.93
	Government trust	3.9	0.95
e	Expert trust	3.68	1.04
Information form trust	Text trust	3.66	0.91
	Picture trust	3.6	0.93
	Video trust	3.72	0.93
	Commentary trust	3.76	0.92
	Real-time interactive trust	4.01	0.89
Information content trust	Environmental information trust	4	0.94
	Input information trust	3.97	0.99
	Certification information trust	3.86	0.97
	Sampling information trust	3.99	0.98
	Credit information trust	3.67	0.98
	Third-party information trust	3.679	0.98
	Sales platform information trust	3.5	1

Consumers have more significant differences in information trust in different presented forms. Text and picture of mainstream quality and safety presentations are challenging to obtain consumer identity (score is 3.65 points and 3.6 points respectively), and more information can be rendered. The score is slightly high at 3.72 points, and commentary trust with certain interactive characteristics has 3.75 points. Live broadcasts have real-time interoperability. Information of interactive features is the highest at 4 points.

The overall level of consumer trust in information subjects is average, and there are significant differences between industry subjects and external subjects: consumers have the highest level of trust in the government with an average score of 3.9, which is basically at a relatively trustful level, followed by experts (3.67), while the news media (3.47) and industry associations (3.43) are next, with lower trust in industry subjects, farmers (3.23 (3.04), enterprises (3.04), and the lowest score for sales platforms (3.39).

From the situation of consumers' trust in information content, information form and information providing subjects, consumers' trust in information content and information presentation form has generally exceeded the trust in information subjects, this implies that with the improvement in consumers' awareness of quality and safety and the accumulation of consumers' knowledge about quality and safety, consumers' trust in their own ability to prevent quality and safety has increased, and are prepared to trust government and experts in addition to relying more and more on their own accumulation of knowledge when making decisions. According to Peng Laqing's (2003) level of lack of public trust, current food safety trust is at the second level [82]. That is, confidence in goods and services and people who provide goods and services is lacking. The degree of trust of outsiders is also low but has not been managed. That is, the government and its management system lose confidence.

### 4.2 Cloglog regression analysis

#### 4.2.1 Regression test

From the results of the parallel line test (see Table 9, Table 10 for the results), the chi-square was 19.18, p > 0.05, and the original hypothesis could not be rejected, considering that the slope coefficient was the same in each response category. From the model fit information, P = 0.00, indicating that at least one has a bias coefficient of one independent variable that is not zero, indicating that the distribution of the data is asymmetric. Since the complementary double logit model is based on the assumption that the error distribution obeys the extreme value distribution, it is applicable to the asymmetric binary discrete data. Therefore, this paper uses the complementary double logit model for regression analysis.

**Table 9:** Parallel line inspection.

Parallel line test^a^				
Model	−2 Logarithm	Bangla	Freedom	Significance
Null hypothesis	1501.367			
Conventional	1482.184	19.184	15	0.206

The original hypothesis indicates that the location parameter (slope coefficient) is the same in each response category.

a Association function: divide the number.

**Table 10:** Model fitting information inspection.

Model fitting information				
Model	−2 Logarithm	Bangla	Freedom	Significance
Only intercept	1616.577			
Finally	1501.367	115.209	15	0.000

Association function: divide the number.

In addition, among the tea consumers surveyed, 836 of them explicitly answered the open-ended question of “willingness to pay for pollution-free tea”, of which 97 (11.6%) were not willing to pay extra premium for pollution-free products (assigned as 1), 331 (39.6%) were willing to pay up to 20% extra for traceable products (assigned as 2), and 408 (48.8%) were willing to pay more than 20% extra for traceable products (assigned as 3). (assigned as 2) were 331 consumers (39.6%), and 408 consumers (48.8%) were willing to pay more than 20% extra (assigned as 3) for traceable products. The dependent variable is an ordered categorical variable with unequal probability of occurrence at the three levels and a higher probability of occurrence at the higher value level, so a complementary double logit model is more appropriate for the analysis.

#### 4.2.2 Model regression results

Table 11 represents the regression results of the complementary double logit model. From the perspective of the regression result, content trust affects customers' willingness to pay. The higher the consumers' content than the certified signal's content, the higher the willingness to pay. Therefore, adding information disclosure aspects, such as place of production, input products, certification processes, and sampling inspection, helps to reduce asymmetrical information between producers and consumers, improving consumers' willingness to pay for pollution-free certification.

**Table 11:** Regression result.

	Estimate	Standard error	Wald	Freedom	Significance	95% Confidence interval	
						Lower limit	Upper limit
[No pollution premium = 1]	−1.269	0.810	2.452	1	0.117	−2.856	0.319
[No pollution premium = 2]	1.038	0.809	1.647	1	0.199	−0.547	2.623
Content trust	0.393	0.077	26.214	1	0.000***	0.242	0.543
Form trust	0.368	0.075	24.160	1	0.000***	0.221	0.514
Third party trust	0.071	0.075	0.916	1	0.338	−0.075	0.217
Industrial subject	0.219	0.071	9.586	1	0.002***	0.080	0.358
Food safety attitude	−0.064	0.086	0.561	1	0.454	−0.232	0.104
Food safety status	−0.009	0.074	0.014	1	0.907	−0.153	0.136
Tea frequency	−0.010	0.055	0.035	1	0.852	−0.118	0.098
Three products a class familiarity	0.220	0.078	8.003	1	0.005***	0.068	0.372
Education level	0.131	0.072	3.342	1	0.068*	−0.009	0.272
Income	−0.040	0.046	0.780	1	0.377	−0.130	0.049
Age	0.009	0.008	1.269	1	0.260	−0.007	0.025
Search	−0.129	0.131	0.970	1	0.325	−0.385	0.127
Experience	0.346	0.084	16.772	1	0.000***	0.180	0.512
Trust	−0.157	0.106	2.187	1	0.139	−0.365	0.051
[Online-oriented = 0]	0.088	0.141	0.390	1	0.532	−0.188	0.364
[Online-oriented = 1]	0a	.	.	0	.	.	.

Association function: divide number.

a. This parameter is redundant.

Formal trust also significantly affects consumers' willingness to pay for pollution-free certified tea. On the basis of improving the existing basic forms such as text and pictures, expanding the presentation of richer forms of certification information, and strengthening real-time communication and interaction with consumers by means of video and visual agriculture can effectively enhance consumers' willingness to pay. At the same time, with the development of online transactions, the time and space separation between consumers and products is more obvious, and thus consumer decisions are more likely to be influenced by product review information. Consumers' trust in industrial subjects significantly affects their willingness to pay for pollution-free certified industries, while the effect of trust in external subjects is not significant. This is because at this stage, consumers have a high level of trust in external subjects, but lack trust in industrial subjects. The industry is the first responsible body for information disclosure and is the holder of a lot of “first-hand quality and safety information”, so if the trust of consumers in the industry can be increased, it will help to realize the premium of certified products. The reason for the insignificant effect of external subject's trust is that there is a significant difference in the level of consumers' overall trust in the outside world, while the extraction of the common factor largely erases the difference between subjects and causes the relationship between them and certification to become insignificant. Among the three attributes, consumers have more experience and will pay extra for certified pollution-free tea. They have to search for product properties and trust attributes and are not likely to pay a premium. This finding is closely related to the popularity of online transactions. The development of online transactions has dramatically expanded the market space of tea consumers and causes time and space separation. Consumers cannot search inline transactions. Judging the quality of tea provides vital information on color, abbreviation, and aroma. Thus, consumers can only rely on brands and reputation to replace search product information, which leads to the significant impact of experience. Regarding the trust of farms and heavy metals, the network transaction amplifies information asymmetry between consumers and producers. The network platform lacks a complete and effective food safety information disclosure mechanism and a sound certification logo supervision mechanism, leading to consumption. The lack of adequate trust in a network platform's certified product causes consumers to pay a premium for the certified product on the network platform.

From other factors, consumers have a high degree of understanding of the three items and are willing to pay higher prices for traceable tea. The three products are not popular among consumers. They will be more fuzzy symbols for consumers. However, passing more information related to the three items is more effective for consumers. Enhancing consumers will also help to improve authentication utility. The higher the degree of education of consumers, the more they are willing to pay more for certified pollution-free tea. The reason is that higher education makes the understanding of food safety issues and quality safety management more accurate and the degree of understanding of certified products higher. Despite the impact, food safety attitude, food safety status, and tea frequency are negatively related to the willingness to pay for tea certification. That is, consumers are concerned. The lower the evaluation of the current quality and safety situation, the fewer consumers are willing to pay the premium for tea certification. Consumers lack confidence in certification.

## 5 Conclusions and policy recommendations

Taking tea as an example, this paper analyzes the current tea consumers' trust in information content, presentation form, subject and other elements of information tools and their influence on pollution-free certified products based on 836 consumer survey data obtained from mobile Internet. It was found that (1) the higher the degree of tea consumers' trust in information content, the higher the additional willingness to pay; (2) form trust also significantly influences tea consumers' willingness to pay for pollution-free certified tea, and the cognitive characteristics of “what you see is what you get” determine the “visible (3) The “what you see is what you get” cognitive feature determines that the “visible” and “interactive” information presentation form can effectively enhance tea consumers' willingness to pay; (4) There is a significant difference in the trust of the subject. The more they care about the attributes of experience goods, the more they know about the three products and one standard, the more educated tea consumers are, the more they are willing to pay higher prices for traceable tea. Based on the above findings, this study believes that to enhance tea consumers' willingness to pay for certified agricultural products and to give full play to the market-driven role of agricultural green transformation, the following three aspects need to be done: (1) focusing on key aspects such as inputs and origin environment, enriching the presentation form of certification information, strengthening intercommunication and interaction with tea consumers by means of video, visual agriculture, etc., and more complete and more intuitive The presentation of quality and safety information, is the primary initiative to enhance the trust of tea consumers. (2) to explore the effective integration of traceability and certification management, the establishment of a more stringent certification mark management system to purify the market environment and enhance the trust of tea consumers in the certification mark itself. (3) Strictly implement the requirements of the first responsible subject of information disclosure of the production and operation subjects, strengthen the quality and safety information disclosure of farmers and enterprises, so that product information, subject credit information and certification information can be effectively integrated to enhance the degree of trust of tea consumers in the industrial subject. At the same time, this study provides personal insights for the development and construction of Anxi Tieguanyin. (1) to create a “cultural circle” of Anxi tea characteristics. By digging deeper into the regional characteristics of Anxi, through short videos, live broadcast and other ways to promote the local traditional craft, the local regional culture will be played and integrated into the tea processing and sales, in addition, to enrich the regional characteristics of tea cultural tourism routes, enrich the content of tea economic and cultural activities, so as to form a diverse regional characteristics of products trusted by consumers. (2) Establish a reliable tea safety traceability system. Anxi government needs to develop a relevant food traceability regulatory system and include tea in the scope of supervision. In addition, tea production and sales enterprises should develop a system of basic data filling, data entry, barcode label printing and pasting work and operator responsibilities, and the relevant leading personnel and functional departments of enterprises should conduct regular inspections of traceability work in order to ensure the normal implementation of the work. (3) Improve the service quality of tea enterprises. For the “reliability” of service quality, tea enterprises should focus on improving the quality of tea products, through the supervision of pesticide residues, access to agricultural products, the traceability of the industry chain, quality inspection of authoritative departments, etc., to enhance consumer confidence in tea products. Of course, there are some shortcomings in this study. For example, some scholars believe that the personality preferences of different groups of tea consumers also affect the willingness to pay for tea consumption, but this paper analyzes the trust of information tools such as information content, presentation form and subject, and does not include consumers' personality preferences in the factors influencing tea consumers' willingness to pay. In the future, the issue of public health certified products, which is of concern to tea consumers, will be explored in more depth, and the research findings will be made more enlightening and the relevant policy recommendations more targeted by enriching the selection of variables and increasing data sources.

## Author contribution statement

Meiying Chen: Analyzed and interpreted the data; Wrote the paper.

Shouxian Huang: Analyzed and interpreted the data.

Guoxing Huang: Contributed reagents, materials, analysis tools or data; Wrote the paper.

Qingqing Dang: Contributed reagents, materials, analysis tools or data.

Kai Li: Conceived and designed the experiments; Performed the experiments.

## Data availability statement

The authors do not have permission to share data.

## Fund project

General project of National Social Science Foundation of China “Research on adaptive Promotion mechanism of Unified Control rule of Featured Agricultural products” (project no.: 20BJY128).

## Declaration of competing interest

The authors declare that they have no known competing financial interests or personal relationships that could have appeared to influence the work reported in this paper.

## Footnotes

1 An information tool is a regulatory tool designed to provide decision-making information to trading entities or regulatory agencies in order to improve the quality of decision making. Key information tools used in food safety risk governance include the information obligations of food production operators, public agency information disclosure systems, incentive systems for information provision, information collection systems and so on.

2 Research products are those that can be fully grasped by consumers before or at the time of purchase, experiential products are those that can only be judged for quality after use, and credence goods are those whose quality cannot be judged even after they have been purchased.
